# Co-occurrence of Ectopic Adrenocorticotropic Hormone (ACTH) Secretion and Syndrome of Inappropriate Antidiuretic Hormone Secretion (SIADH) in Small Cell Lung Cancer: A Case Report

**DOI:** 10.7759/cureus.92128

**Published:** 2025-09-12

**Authors:** Zhan Rong, Anisha Mahat, Jordan Barnett Kradjian, Viraj Modi, Imran Baig

**Affiliations:** 1 Internal Medicine, Stony Brook University Hospital, Stony Brook, USA; 2 Internal Medicine, Northport Veterans Affairs (VA) Medical Center, Northport, USA

**Keywords:** ectopic cushing syndrome, hypokalemia, hyponatremia, paraneoplastic syndrome, small cell lung cancer, small cell lung cancer induced-siadh

## Abstract

Small cell lung cancer (SCLC) is a neuroendocrine malignancy often associated with paraneoplastic syndromes, such as syndrome of inappropriate antidiuretic hormone secretion (SIADH) and ectopic Cushing’s syndrome (ECS). While each is individually recognized, their concurrent presentation is exceptionally rare and poses diagnostic and management challenges. A 49-year-old male with a complex medical history presented with back pain, profound hyponatremia (sodium 119 mmol/L), and hypokalemia (potassium 2.5 mmol/L). Imaging revealed a large right hilar mass with mediastinal lymphadenopathy and hepatic lesions. Biopsy confirmed high-grade neuroendocrine carcinoma consistent with SCLC. Persistent electrolyte abnormalities and treatment-resistant hypertension prompted an endocrinologic workup, revealing elevated cortisol (> 64 μg/dL) and adrenocorticotropic hormone (ACTH) (377 pg/mL), consistent with ectopic ACTH production. Concurrent SIADH was diagnosed based on low serum osmolality, high urine osmolality, and urine sodium > 30 mmol/L. The patient was treated with carboplatin and etoposide as per oncology recommendations. Management of SIADH and ECS was challenging due to the complex interplay of fluid retention, cortisol excess, and risks associated with vasopressin antagonists and cortisol-lowering agents. Chemotherapy was prioritized to address the underlying tumor and paraneoplastic processes. This rare case of concurrent SIADH and ECS in SCLC underscores the importance of early recognition and multidisciplinary management. Awareness of overlapping paraneoplastic syndromes is critical for timely diagnosis and effective treatment, which can prevent life-threatening complications and improve outcomes.

## Introduction

Small cell lung cancer (SCLC) is an aggressive neuroendocrine tumor that accounts for up to 15% of lung cancer diagnoses [1]. It is often associated with myriad paraneoplastic syndromes, which manifest as tumor cells producing ectopic hormones, cytokines, or other substances that trigger various outcomes [2].

The most common well-documented paraneoplastic syndrome is the syndrome of inappropriate antidiuretic hormone secretion (SIADH). The abnormal excessive secretion of antidiuretic hormone (ADH) by cancer cells causes increased water retention and dilutional hyponatremia [3]. SIADH occurs in 10-45% of patients with SCLC [2,4]. The second most common paraneoplastic manifestation is ectopic adrenocorticotropic hormone (ACTH) production, which can lead to ectopic Cushing’s syndrome (ECS). The ectopic ACTH secretion causes a dysregulation of the negative feedback system, leading to overproduction of cortisol [5]. Accordingly, classic Cushingoid clinical features such as weight gain, hypertension, hypokalemia, and central obesity can be found, but many patients present with non-specific symptoms such as weakness and mild electrolyte disturbances [6]. ECS occurs in about 1-5% of patients diagnosed with SCLC [2,6]. Despite the individual endocrine syndromes being common in SCLC, their co-occurrence is exceedingly rare per literature review, with only 10 previously reported cases [7]. Not only is this co-occurrence exceedingly rare, but it is also difficult to diagnose and treat, as ACTH and ADH have opposing endocrine effects, leading to diagnostic ambiguity and treatment dilemmas [7].

This report highlights an incredibly rare case of concurrent SIADH and ectopic ACTH in a patient with newly diagnosed SCLC. It highlights the unique challenges in the diagnosis and management of SCLC with multiple concurrent paraneoplastic syndromes and emphasizes the need for physicians to be aware of the possibility of such co-occurrences.

## Case presentation

The patient is a 49-year-old male with a past medical history of multiple sclerosis (MS) complicated by double vision, traumatic brain injury complicated by short-term amnesia, chronic obstructive pulmonary disease (COPD), current heavy smoking (two packs per day for 41 years), anxiety, and a history of polysubstance use. The patient initially presented with back pain and was found to have a new T11 compression fracture on a thoracic spinal X-ray. The patient also reported chronic nausea and new lower extremity edema. The initial presentation was also notable for hypertension, serum sodium of 119 mmol/L, serum potassium of 2.5 mmol/L, platelet count of 74 K/uL, and elevated total bilirubin. 

**Figure 1:**
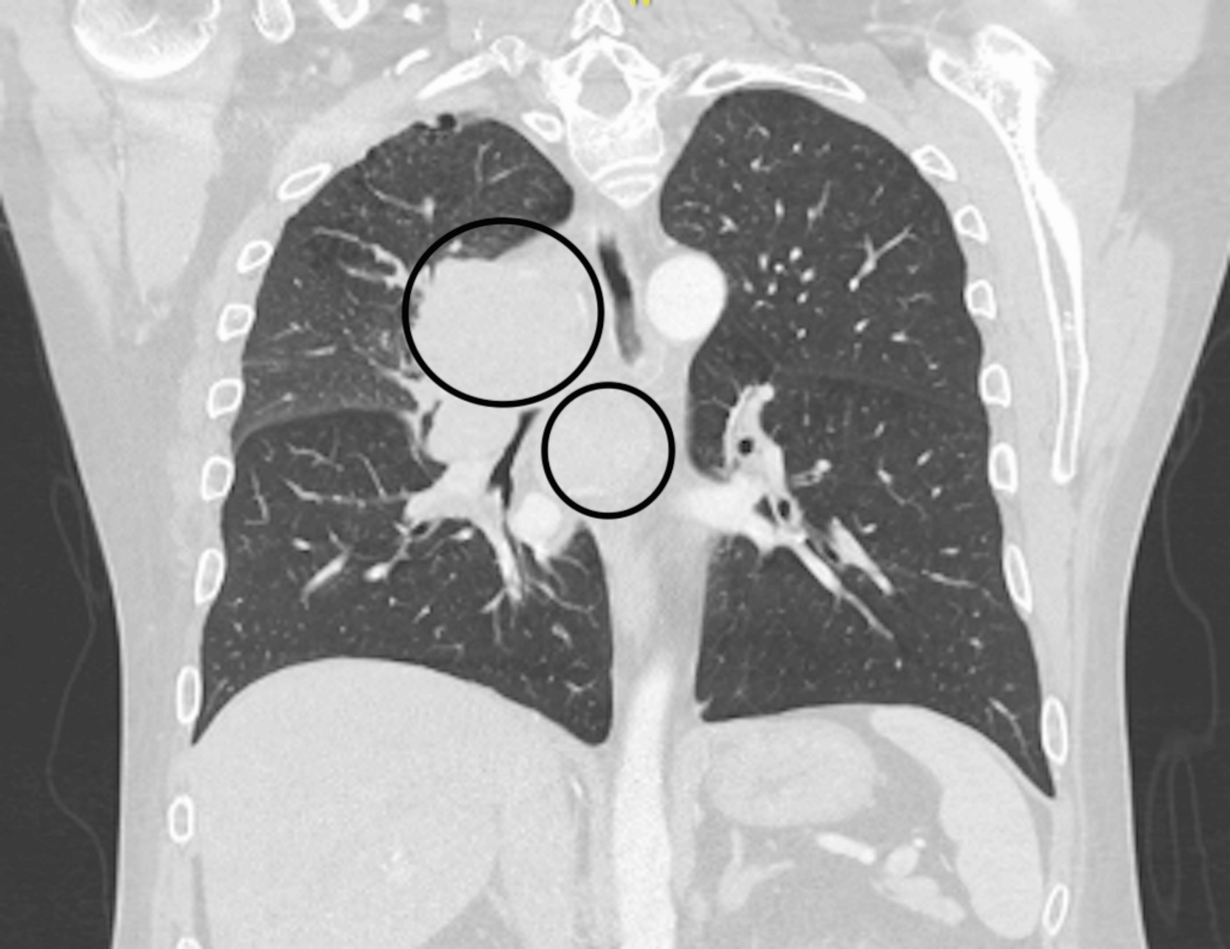
CT chest showing bulky right hilar and subcarinal lymphadenopathy. The right hilar mass and subcarinal lymphadenopathy have been marked using open circles.

**Figure 2:**
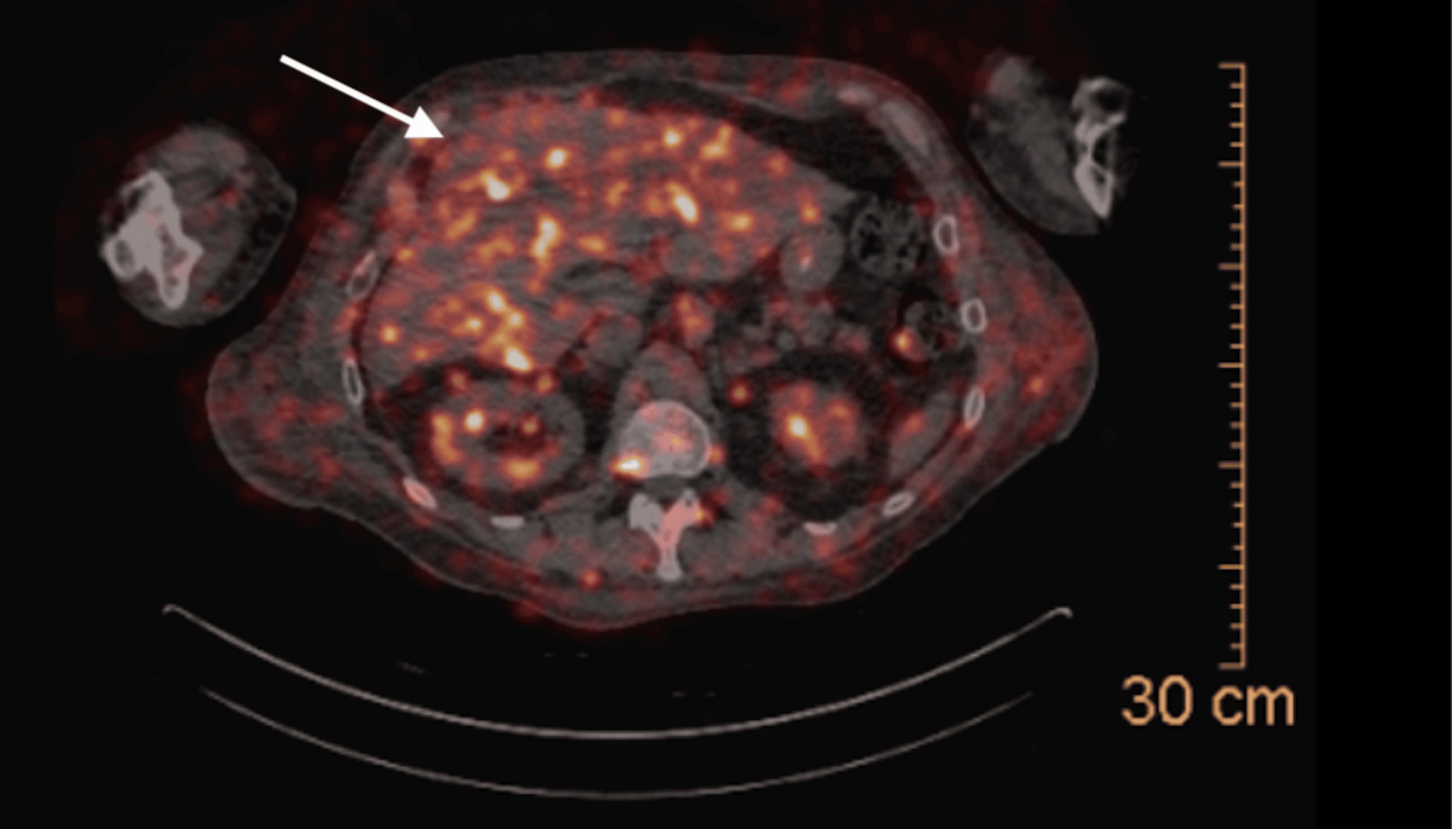
PET-CT showing FDG tracing in the heterogeneous liver. Arrow points toward the fluorodeoxyglucose (FDG) signals seen in the liver.

Notably, three weeks prior to his presentation, the patient was incidentally found to have a new hilar lung mass on chest X-ray. A CT scan performed at the time demonstrated enlarged, bulky lymphadenopathy of the right paratracheal, subcarinal mediastinal regions, and right hilum with marked extrinsic compression of the right main stem bronchus with numerous adjacent peribronchiolar nodular opacities, with the largest measuring up to 2.3 cm x 1.3 cm (Figure 1). On the day of the presentation, the patient underwent a PET-CT, which exacerbated his back pain and prompted his visit to the emergency room. The available PET-CT imaging was significant for a bulky right hilar lymphadenopathy measuring up to 11.8 cm x 7.5 cm x 11.0 cm, as well as a mildly enlarged heterogeneous liver with questionable fluorodeoxyglucose (FDG) avidity (Figure 2). Due to pain, the patient was not able to tolerate the full FDG phase of the study, resulting in the absence of FDG tracing from the lower chest and above.

**Figure 3:**
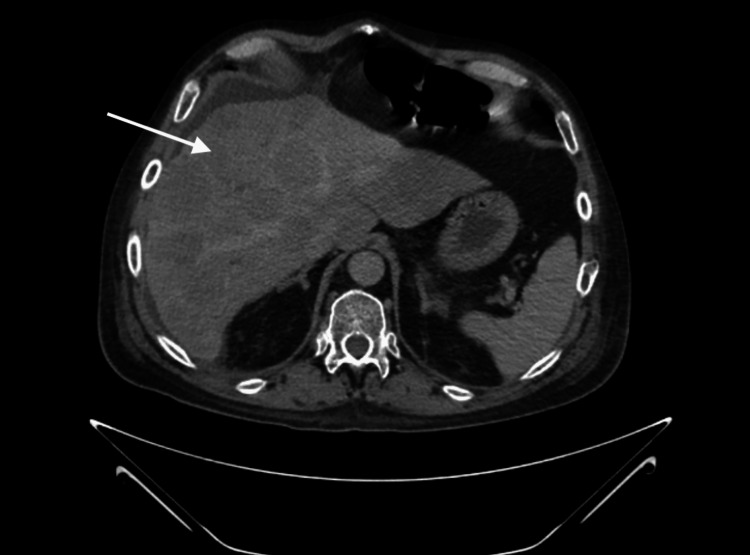
Repeat CT abdomen showing heterogeneous liver with multiple masses concerning for metastasis. Arrow points towards the heterogeneous masses seen in the liver on CT.

**Figure 4:**
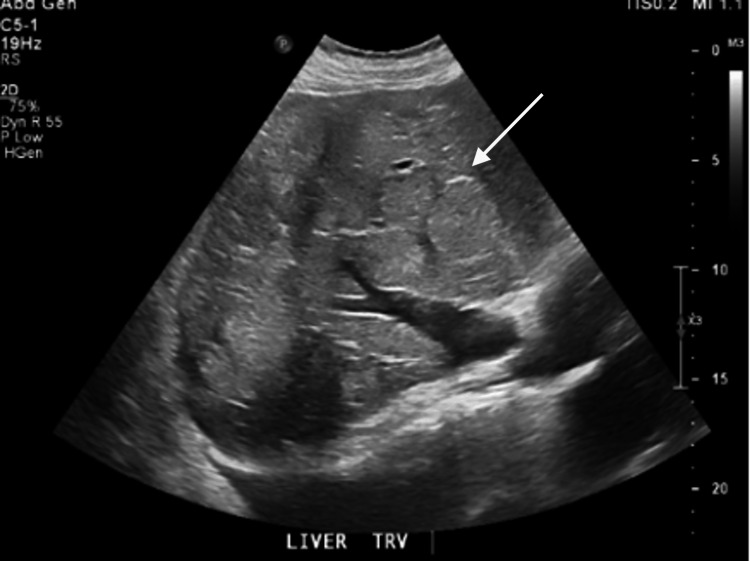
Abdominal US showing heterogeneous echotexture with a nodular contour. Arrow points towards the heterogeneous liver and masses.

**Figure 5:**
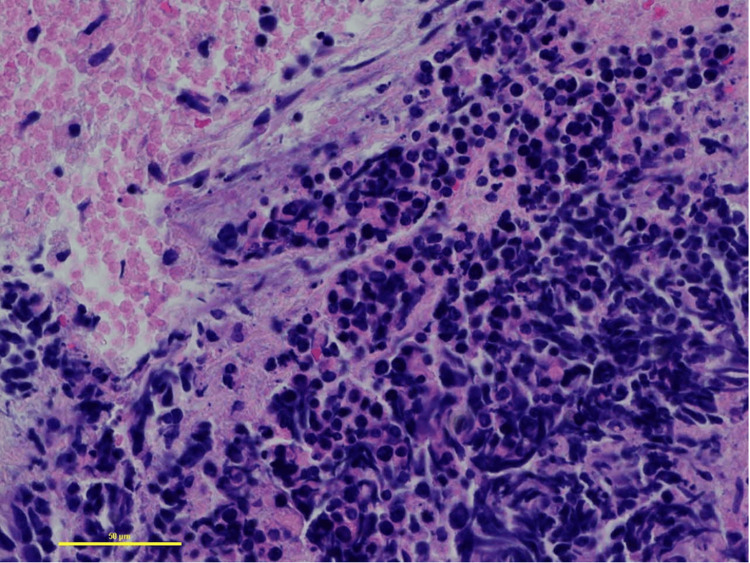
Pathology slide of lung mass showing SCLC. This slide is 400x magnification, showing tumor cells with necrosis, crush artifact, and scant cytoplasm. Immunostains showed positive staining with TTF1 and CD56, with up to 80% Ki-67 labeling. SCLC: small cell lung cancer

During the admission, repeat abdominal CT imaging showed similar findings, including a lung mass, multiple liver masses, and concern for cirrhosis (Figure 3). Abdominal ultrasound showed heterogeneous echotexture to the liver with a nodular contour, potentially consistent with cirrhosis, in addition to at least one mass-like lesion within the right hepatic lobe measuring 6.0 cm and likely additional masses throughout the hepatic parenchyma partially visualized (Figure 4). The infectious and autoimmune workup for cirrhosis was unremarkable. On hospital day two, pathology from the lung biopsy returned with high-grade neuroendocrine carcinoma consistent with small cell carcinoma of the lung (Figure 5). The hospital course was complicated by persistent hypertension, hyponatremia, and hypokalemia, despite adequate back pain control, aggressive antihypertensive treatment, fluid restriction, and electrolyte repletion. The patient continued to maintain potassium levels as low as 2.8 mmol/L despite aggressive repletion. In addition, his magnesium level remained above 2 mg/dL throughout his admission at the Northport Veterans Affairs (VA). The patient was started on spironolactone to help modulate the elevated blood pressure and hypokalemia. An etiology of ectopic ACTH production was hypothesized as a potential explanation. An endocrinologic workup was obtained, which subsequently showed suppressed aldosterone of <1 ng/dL, normal plasma renin activity of 0.497 ng/mL/hr, a severely elevated morning cortisol level of >64 ug/dL, and a severely elevated ACTH level of 377 pg/mL. Notably, an MRI two months prior to admission for evaluation of MS showed no pituitary mass. A summary of the patient’s admission vital signs and diagnostic labs is included in Table 1.

**Table 1:** Electrolyte trends and key laboratory findings. ↓=below reference range; ↑=above reference range; ↑↑=severely elevated above reference range; ↓↓↓=lowest value during hospital course; ↑↑↑=maximum value during hospitalization; "-" = not measured or not clinically relevant at that time point.

Parameters	Reference normal range	Initial presentation	Hospital course	Endocrine workup	Hospital day 7	Readmission
Serum sodium (mmol/L)	135-145	119↓	-	-	138	118↓
Serum potassium (mmol/L)	3.5-5.0	2.5↓	2.8↓↓↓	-	3.7	
Platelet count (K/uL)	150-400	74↓	-	-	-	-
Total bilirubin (mg/dL)	<1.2	3.3	-	-	-	-
Magnesium (mg/dL)	1.7-2.2	-	>2	-	-	-
Aldosterone (gm/dL)	3.0-16.0	-	-	<1 ng/dL	-	-
Plasma renin activity (ng/mL/hr)	0.2-1.6	-	-	0.497	-	-
Morning cortisol (ug/dL)	5.0-25.0	-	-	>64↑↑	-	-
Adrenocorticotropic hormone (pg/mL)	7-63	-	-	377↑↑	-	-
Serum osmolality (mOsm/kg)	275-295	-	-	259↓	-	-
Urine osmolality (mOsm/kg)	50-1200	-	-	518	-	-
Urine sodium (mmol/L)	20-50	-	-	51↑	-	-
Systolic blood pressure (mmHg)	90-130	148	214↑↑↑	-	-	-
Diastolic blood pressure (mmHg)	60-80	92	112↑↑↑	-	-	-
Heart rate	60-100	64	-	-	-	-
Temperature (degrees Fahrenheit)	97-99	98.8	-	-	-	-

**Figure 6:**
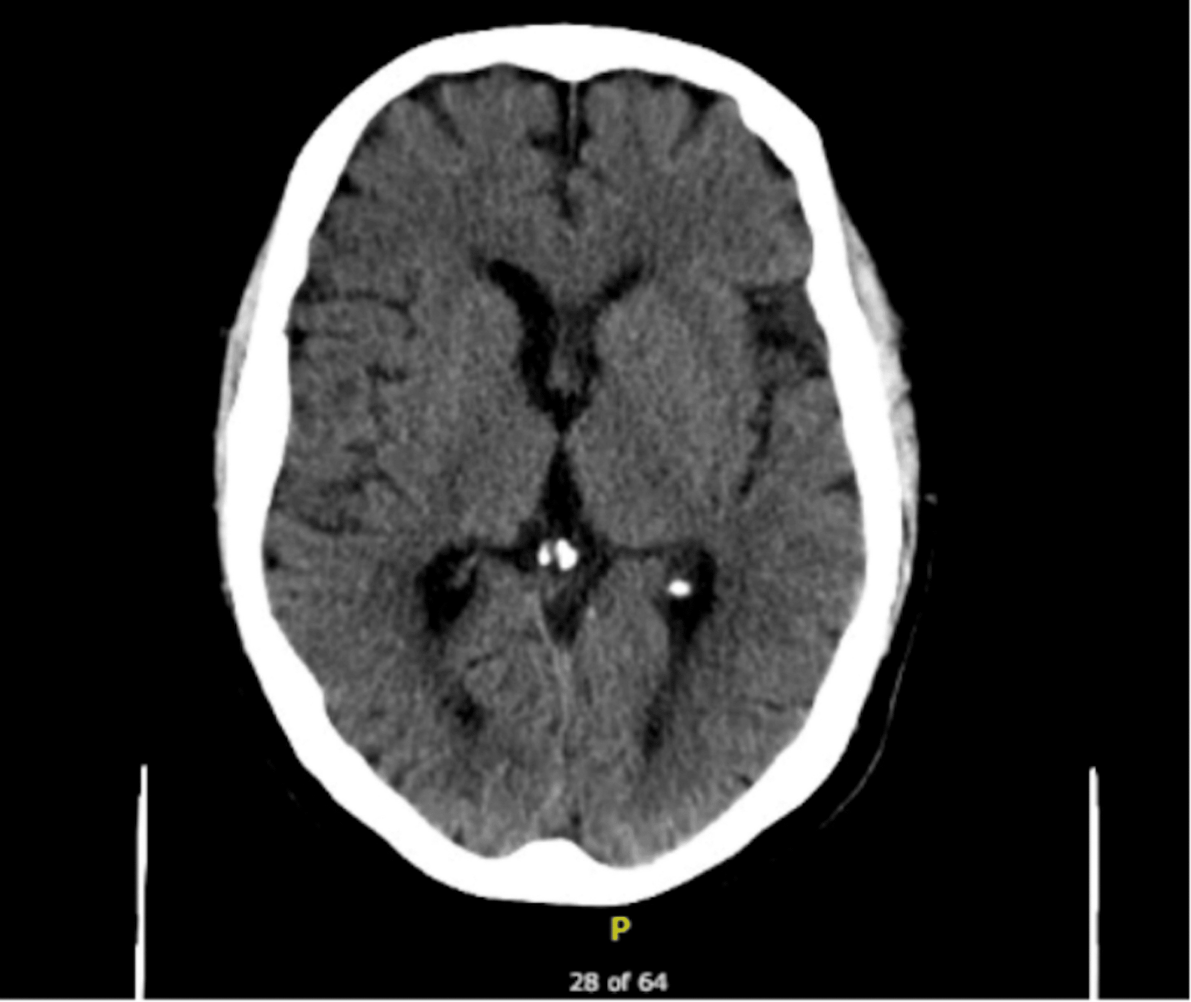
CT head showing no acute intracranial pathology that would explain seizure activities.

**Figure 7:**
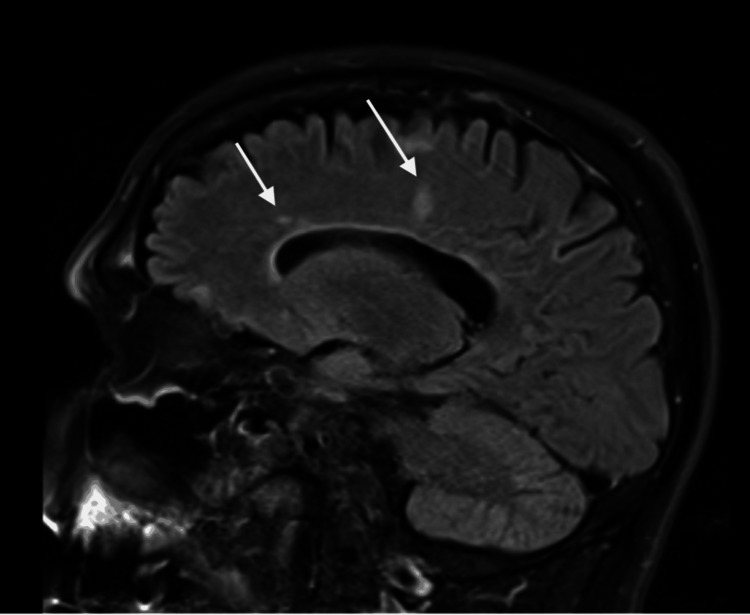
MRI brain showing findings consistent with MS but no evidence of metastasis. Arrows point to the findings consistent with multiple sclerosis (MS).

**Figure 8:**
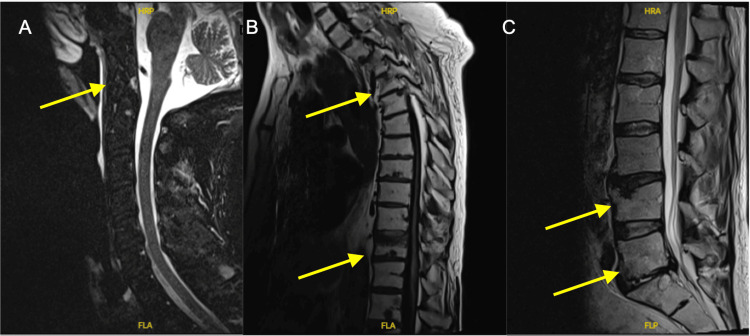
MRI spine images showing lytic lesions marked with yellow arrows. A: cervical spine lesions; B: thoracic spine lesions; C: lumbar spine lesions

On hospital day six, the patient was started on carboplatin and etoposide treatment for SCLC. On hospital day seven, the patient was found to have seizure-like activity on examination with a possible post-ictal state. The CT head showed no acute hemorrhage or infarct, with the only significant finding of a focal mucosal cyst in the right maxillary sinus (Figure 6). Serum electrolytes were overall normal at the time of his seizure. Sodium was 138 mmol/L, and potassium was 3.7 mmol/L. Given high suspicion for seizure, the patient was transferred to the medical oncology unit at the VA-affiliated University Hospital for electroencephalogram (EEG) monitoring as well as continued SCLC treatment. The patient was subsequently started on seizure prophylaxis with levetiracetam. Video EEG revealed no obvious seizure activity, and an MRI of the brain showed no metastasis (Figure 7). The patient’s home urea was increased, which improved the patient’s sodium. MRI of the cervical, thoracic, and lumbar spine showed lytic lesions at C2, C4, T1, L1, S1, S2, and bilateral iliac bones consistent with metastatic disease (Figure 8). Neurosurgery recommended no surgical intervention, and the patient was discharged 14 days after transfer.

Two weeks after discharge, the patient was readmitted to the University Hospital for pneumonia and severe hyponatremia with a sodium of 118 mmol/L. Hyponatremia was felt to be due to SIADH in the setting of SCLC and concomitant infection, which subsequently improved after use of hypertonic saline infusion. While in the hospital, he received the second cycle of carboplatin and etoposide. The patient was then discharged after stabilization of sodium and resolution of pneumonia.

The patient returned to the University Hospital one week after his most recent discharge and was found to have post-obstructive pneumonia, with resultant septic shock requiring intubation. Despite aggressive measures, the patient continued to deteriorate, and the decision to pursue comfort care was made by the next of kin. The patient passed away shortly after withdrawal of life-supporting measures.

## Discussion

ECS is a disorder of overproduction of ACTH occurring in a tumor originating outside of the pituitary gland. Increased ACTH causes downstream effects, including treatment-resistant hypertension, hypercortisolism, hypokalemia, and muscle weakness. Our case involves a patient with treatment-resistant hypertension, confirmed elevated ACTH production, hypercortisolism, and recent brain imaging with normal pituitary findings. Studies suggest that ECS is underdiagnosed and occurs in 1-5% of patients with SCLC, making it quite rare [6]. Similarly, ectopic ADH secretion can occur in the setting of lung cancer, resulting in hyponatremia and its associated symptoms such as confusion, headache, nausea, vomiting, and muscle cramps. This patient was being treated for chronic hyponatremia with urea and fluid restriction for more than two months prior to the onset of his acute back pain. Therefore, his hyponatremia was likely due to SIADH secondary to SCLC and unrelated to the recent onset of refractory pain.

Studies suggest that nearly half of all ECS are attributable to bronchial carcinoid or SCLC tumors [8]. Studies also suggest that SIADH production by tumor cells is the most common cause of hyponatremia in patients with lung neoplasms [9]. Although ectopic ACTH and ADH syndromes are each known to occur in patients with SCLC, their simultaneous occurrence is incredibly rare and cited in very few studies.

The management of concurrent ECS and SIADH is challenging, as both syndromes and their effective therapies can reciprocally exacerbate each other. Firstly, excessive cortisol production can mask SIADH given its weak mineralocorticoid activity [10]. The excessive cortisol saturates 11-beta-hydroxy-steroid-dehydrogenase, which normally metabolizes cortisol to cortisone, which has no mineralocorticoid activity [11]. Secondly, management of SIADH with fluid restriction can further increase cortisol production, likely due to the body’s perception of dehydration as stress [12,13]. Finally, the choice of medication to treat ECS must be selected carefully, given that many have undesirable adverse effects and may even precipitate SIADH. Therapeutic agents such as ketoconazole, which is used to control excess cortisol, have been reported to increase SIADH incidence to 44% when used in conjunction with vincristine, a common agent used to treat SCLC [14]. In addition, vasopressin receptor inhibitors such as conivaptan and demeclocycline have limited evidence in treating SCLC-induced SIADH. Vasopressin receptor inhibitors increase the risk of overly rapid sodium correction, leading to osmotic demyelination syndrome, while demeclocycline has a delayed onset of action and nephrotoxicity. Finally, these agents do not treat the cause and source of SIADH, which is the SCLC.

The optimal therapy for this patient with primary SCLC is chemotherapy and possible radiation therapy. This treatment would be expected to remedy the paraneoplastic syndromes as well. As an adjunct, symptomatic control can also be achieved with medical therapy for hypercortisolism, such as ketoconazole, metyrapone, osilodrostat, and mitotane, in addition to therapy for SIADH, such as fluid restriction and sodium repletion.

According to literature reports, SIADH is a negative prognostic factor of mortality in patients with lung tumors [9]. This patient’s concurrent seizures and neurological medical history portend an even worse prognosis, which contributed to his eventful clinical course and poor outcome.

## Conclusions

We present a case of SCLC with rare concurrent paraneoplastic syndromes of ectopic ACTH and SIADH. Our case demonstrates the challenges and significance of recognizing these syndromes early to help guide proper diagnosis and management. In addition, the co-occurrence of these syndromes further adds to the complexity when considering potential treatment options. Careful monitoring and a multidisciplinary approach are important to balance the management of excess cortisol, fluid status, and electrolyte abnormalities. Prompt diagnosis and management can help prevent life-threatening complications and improve patient care. Further research is encouraged to help develop strategies in the care of patients with multiple paraneoplastic syndromes.
